# The Question‐prompt list (QPL): Why it is needed in the Indian oncology setting?

**DOI:** 10.1002/cnr2.1316

**Published:** 2020-12-09

**Authors:** Shweta Chawak, Mahati Chittem, Sravannthi Maya, Haryana M. Dhillon, Phyllis N. Butow

**Affiliations:** ^1^ Department of Liberal Arts Indian Institute of Technology Hyderabad Hyderabad India; ^2^ Centre for Medical Psychology & Evidence‐based Decision‐making, School of Psychology The University of Sydney Sydney New South Wales Australia

**Keywords:** India, oncology, patient–caregiver, question prompt list, question‐asking

## Abstract

**Background:**

In India, caregivers are an integral part of the illness experience, especially in cancer, to the extent that they can become proxy decision‐makers for the patient. Further, owing to acute resource constraints in the Indian healthcare system, it may be difficult for oncologists to assess and elicit questions from each patient/caregiver. Consequently, there is a need to address these unique aspects of oncology care in India to improve patient outcomes and understanding of their illness and treatment. This can be achieved through a Question Prompt List (QPL), a checklist used by care recipients during medical consultations.

**Recent Findings:**

This narrative review will first introduce research on the development and effectiveness of the QPL, and then it will highlight current gaps in oncology care in India and explore how the QPL may aid in closing these gaps.

A literature search of the empirical research focused on the development, feasibility and acceptability of the QPL in oncology settings was conducted. The final review included 40 articles pertaining to QPL research. Additionally, psycho‐oncology research in India centered on information needs and experiences was reviewed.

Current Indian psycho‐oncology research reports patients' want to be actively involved in their cancer care and a need for more illness information. However, a high demand on physicians' resources and the family caregivers' interference can be barriers to meeting patients' information/communication needs. International research demonstrates that a QPL helps structure and decrease consultation time, improves patient satisfaction with care, and improves the quality of communication during medical encounters.

**Conclusion:**

QPLs for Indian patients and caregivers may focus on the scope of medical consultations to address patient needs while influencing the course and content of the patient‐caregiver‐physician interactions. Further, it can address the resource constraints in Indian oncology care settings, thus reducing the physician's burden.

## 1 BACKGROUND

A fundamental way in which patients and family members can participate in medical consultations is by asking questions, enabling their information needs to be met.^1^ Question‐asking is associated with greater information delivery, fewer unmet needs for information, and better patient recall.^2^, ^3^ Although patients have a need to seek information regarding their illness, they encounter several barriers to finding and consuming this information.^4^, ^5^ For example, Datta et al^4^ found patients indicated a need for information but were unable to convey it to the physician due to time constraints, fear of asking questions, family reluctance, and feelings of incompetence. To help patients overcome these barriers to question‐asking during medical consultations, research has examined the efficacy of techniques such as tailored education coaching,^6^ communication‐centered interventions such as consultation planning,^7^ and decision boards.^8^

One such tool used for promoting question‐asking is the Question Prompt List (QPL).^1^ A QPL is a list of questions organized in categories patients may like to ask about their illness. Questions are derived from interviews with patients, family members/caregivers, and healthcare professionals. The QPL is provided to the patient before the consultation and patients encouraged to think about the most important questions they would like to ask during their upcoming consultation. The QPL can be used either as an individual intervention^9^ or be paired with other interventions (eg, communication skills program).^10^ Although QPLs have been mainly implemented with patients,^9^, ^11^, ^12^ one study used a QPL with patients and caregivers.^3^ In psycho‐oncology research, QPLs have been used during various stages of the patient's illness and treatment including in first consultation, before surgery, when asked to participate in a clinical trial, and when cancer has advanced.^1^, ^3^, ^9^, ^11^, ^12^ The QPL was observed to help in overcoming patients' inhibitions in asking questions,^2^, ^3^, ^13^ provided structure to the medical consultation,^13^ increased information given to patients,^2^ and improved recall.^2^, ^13^

In India, there are vastly fewer physicians than patients (ratio 0.77:1000),^14^ which places a huge demand on physicians' availability and time which, in turn, hinders patients' access to quality healthcare.^15^ Given this, it may be effective to empower patients with the skills to structure communication to obtain the information and support most relevant to their situation. This review aims to (i) introduce empirical research pertaining to the development and effectiveness of the QPL and (ii) justify the use of the QPL in the contexts of Indian cancer care.

## 2 METHODS

### 2.1 Search strategy

A narrative literature review as recommended by Ferrari^16^ and Green et al^17^ was conducted to understand the development, feasibility, and effectiveness of the QPL. A database search was carried out in Web of Science, PubMed, and Google Scholar for articles published from 1994‐2020. The following words/terms were used to perform this search: (i) question prompt list, (ii) question prompt sheet, (iii) prompt list, (iv) prompt sheet, (v) development, (vi) acceptability, (vii) feasibility, (viii) pilot, (ix) random controlled trial (x) RCT, (xi) oncology, and (xii) cancer, and Boolean operators of “and” and “or” were used to combine the above words/terms such as (i) and (v), (iii) and (ix), and (xi).

### 2.2 Selection criteria

**FIGURE 1:**
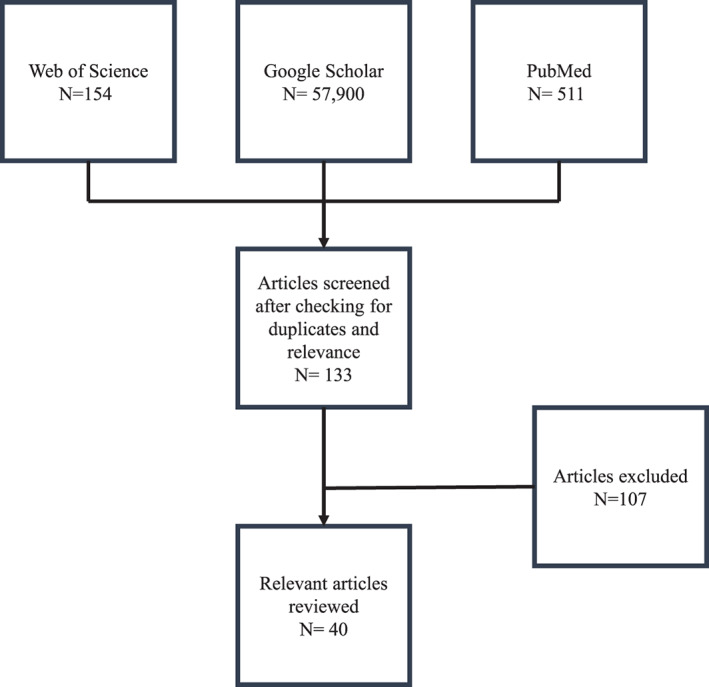
Flowchart of the Literature selection process for the present article

The selection criteria were original and empirical research which presented the development, acceptability, and/or effectiveness of the QPL in oncology settings. Exclusion criteria were articles which were: (i) implementing other interventions (eg, References 18, 19) paired with a QPL, (ii) existing QPLs adapted in different languages (eg, References 20, 21), (iii) QPL studies conducted in other illnesses (eg, References 22, 23, 24, 25), and (iv) review articles (eg, Reference 26). Overall, 58 565 articles were identified in the initial screening. On the basis of relevance to and fulfillment of the selection criteria, 40 studies were taken into consideration for this review (see Figure 1). These articles are as follows: (i) development of the QPL (n = 15), (ii) feasibility acceptability of the QPL (n = 11), and (iii) effectiveness of the QPL (n = 14).

### 2.3 The Question Prompt List: an overview

**FIGURE 2:**
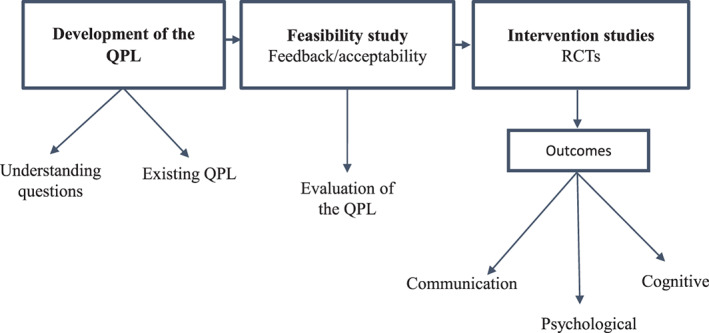
Three phases of research carried out to develop and test the Question prompt list

The three stages of QPL research are represented in Figure 2, and a detailed description is provided below:

#### 2.3.1 Development of the QPL

The initial stage of developing a QPL includes conducting a qualitative study such as a focus group discussion (FGD) (eg, References 27, 28), individual interviews (eg, References 29, 30), expert panels (eg, References 31, 32, 33), or using a Delphi method (eg, References 34, 35, 36) to understand the specific questions that could usefully be asked by the target population regarding their illness and/or treatment. A few studies used previously published QPLs which were reviewed and modified by a panel (ie, patient, family caregivers, and healthcare professionals).^31^, ^35^, ^37^ Through these qualitative methods and subsequent data analyses, a draft list of questions is generated for the patient/caregiver to use at the treatment consultation. The list can comprise questions and concerns the patient (i) would like to discuss with the healthcare professional (often physician) and (ii) that were not addressed in previous consultations. The list can be grouped into separate categories such as questions/concerns about the diagnosis, treatment, financial aspects, and timeline.

As questions patients may have can vary according to the type and stage of the illness and treatment,^1^, ^3^, ^9^, ^11^, ^12^ QPLs have been developed for specific oncology‐related illness and treatment contexts: type of cancer such as esophageal,^31^ breast,^34^ and brain,^37^; for patients in a surgical setting or undergoing chemotherapy^38^; patients being invited to participate in clinical trials^27^; when receiving outpatient palliative care^35^; and to facilitate end‐of‐life discussions and advance care planning among patients with an advanced cancer.^32^ More recently, a QPL was developed for family members of paediatric palliative care patients.^36^ Table 1 provides an overview of studies on the development of the QPL.

**TABLE 1:** Overview of studies on development of the Question‐prompt list (QPL)

Development of a QPL					
Study	Country	Topics of QPL	Methodology	n	Broader topics covered within QPL
Brown et al^33^	Australia	Medical oncology	Content analysis of 20 taped consultations	2 (Med. Onco) 2 (Psych)	Diagnosis, Tests, Treatment, Prognosis, Psychosocial issues & Support services available
Bruera et al^39^	USA	Breast C	Existing QPL† Expert panel‡	‐	Diagnosis, Treatment, Prognosis
Clayton et al^29^	Australia	Palliative care	FGDs and Individual interviews	19 (P) 24 (C)	PC Team & Service, Lifestyle & Qol, Treatment, Illness & expected future, Support, EOL Issues
McJannett et al^28^	Australia	Surgical Oncology	FGDs	22 (P)	Preliminary negotiations & Diagnosis, Further investigations & choice of medical professional & Second opinion, Treatment information, Support
Albada et al^34^	Netherlands	Genetic testing	Expert panel^§^	8	Hereditary, Genetic counselling, Being a carrier, Breast C risk, Emotional consequences, Consultation
Brown et al^27^	USA	Clinical trial (Lung, breast, prostate cancer)	FGDs	20	Understanding choice, Benefits & Risks, Procedure, Conflict of interest, Alternative treatment
Lim et al^10^	Singapore	Surgical onco (Abdomen and breast)	Complied by the researchers	‐	Diagnosis, Operation & post operation care, Treatment, Lifestyle change
Shirai et al^40^	Japan	Advanced C	Existing QPLs¶ Interviews	14(P) 5 (Onco)	Diagnosis, Symptom, Test, Treatment, Life, Family, Psychological issues, Prognosis, Other issues
Smets et al^31^	Netherlands	Esophageal C.	Translated to Dutch Questions added	‐	Diagnosis, Tests, Prognosis, Treatment options, Multidisciplinary team, Surgery, Effects of surgery, Qol, Support information
Langbecker et al^37^	Australia	Brain tumor	Thematic analysis of existing QPL	‐	Diagnosis, Prognosis, Symptoms & changes, Treatment, Support, After treatment, Health professional team
Walczak et al^32^	Australia and USA	Advanced C	Expert Panel analyzed	7 (HCR and HP from USA and Australia)	Diagnosis, Treatment, Making decisions, Future expectation, EOL, Family concerns
Eggly et al^38^	USA	Racial disparity	Group & individual meeting (RAC)	6	Diagnosis, Treatment, Side‐effects, Goal of Treatment & Prognosis, Support service, Treatment schedule
Arthur et al^35^	USA	Palliative care	Delphi Method (expert panel)	22 (Palliative physician and midlevel providers)	Treatment, Symptom, Carer‐related, EOL, Palliative services, Support
Rodenbach et al^30^	USA	Palliative care	Existing QPL# FGDs & Individual interviews	19 (P)	Illness‐related, Treatment, Illness management, Prognosis, Lifestyle change, EOL, Support, Others: Financial, Test results, Vacation planning
Ekberg et al^36^	Australia	Paediatric Palliative care	Delphi Method (Expert panel and P's Family members	29 (Experts) 7 (P's family members)	Seven domains

#### 2.3.2 Feasibility/acceptability of the QPL

Once the QPL is developed, its feasibility and acceptability are assessed. Data can be collected at three time points: (i) before the medical consultation (ie, responses of the patient/caregiver during the introduction of the QPL), (ii) during the medical consultation (ie, recording/observing the dynamic interaction with and feedback from the healthcare professional), and (iii) post consultation (ie, obtaining reflective feedback about the QPL from the patient/caregiver). Previous research collected some psychological data during these phases including patient satisfaction, levels of anxiety, and depression.^11^, ^12^, ^31^, ^41^ In most studies, patients reported the QPL to be useful, such that it could be beneficial for the caregivers to engage in question‐asking,^31^, ^32^ it was a good communication tool which organized and prompted patients to ask questions during the consultation,^38^, ^42^ helped patients address critical questions related to end‐of‐life, and reduced the burden of comprehending excessive information.^32^, ^43^ Similarly, McLawhorn et al^44^ reported that the use of QPL increased the number of do‐not‐resuscitate orders and hospice referrals. During the consultation, it was observed that patients using the QPL did ask more questions,^31^ were able to address important concerns they had,^45^ and engaged in a meaningful discussion with their physician,^27^, ^29^ and the time taken for the medical consultation was not impacted.^29^, ^31^ Interestingly, the QPL helped patients discuss delicate and difficult topics such as prognosis related questions.^29^, ^31^ On the other hand, it was noted that some topics were not addressed by patients during the consultation such as sexuality, body image, spirituality, and psychosocial support.^29^, ^31^ Yet, a recently implemented QPL reported to have increased overall treatment knowledge among the patient,^46^ thus underscoring the role a QPL can play in knowledge acquisition, In the postconsultation feedback, patients reported reduced anxiety,^29^, ^41^ and that they did not find the QPL questions distressing,^39^ while their levels of satisfaction with the consultation remained unchanged.^31^ Likewise, during post consultation, clinicians reported the QPL was useful in addressing sensitive topics and reported need for such tool.^42^, ^43^ Table 2 provides an overview of studies understanding the feasibility of the QPL.

**TABLE 2:** Overview of the studies understanding the feasibility of the QPL

Study	Country	n	Utility of the QPL		
			Preconsultation	During consultation	Postconsultation/Follow‐up
Clayton et al^29^ Palliative care	Australia	23	95% QPL useful; 80% helped verbalize queries and concerns	↑ Prognosis Q. Consultation time, no effect	↓ Anxiety 100% QPL useful; 81.82% verbalized queries and concerns
Glynne‐Jones et al^45^	UK	254			65% QPL useful 61.41% information was about right 84.67% Asked more questions
Dimoska et al^43^ Medical Surgical Radiation Palliative	Australia	P (139) Cl‐Pre (20) Cl‐Po (10)	30% Cl‐Pre: QPL make communication easy All Cl‐Pre: help initiate discussion		92% P: very or fairly useful 27% P: definitely use it again 53% probably use it again 60% Cl‐Po: initiate sensitive topics 90% Cl‐Po: need such communication tool
Brown et al^11^ Clinical Trial	USA	20	87% Preferred to know 18.7 average Q (wanted to ask) ↑ need to ask question		↑ Treatment related Q.
Langbecker et al^37^ Brain Tumour	Australia	20			↑ Usability, ↑ Ease in asking
Smets et al^31^ Esophageal Can.	Netherlands	18 (IG) 12 (CG)	19/38 QPL questions marked in the QPL: ↓ Q. on psychosocial care from QPL ↑ Q. on treatment and diagnosis from QPL	↑ Q. asking by IG(vs CG) ↑ prognosis Q. by IG(vs CG) Consultation length; *ns*	Patient satisfaction; IG(vs CG), *ns* 11/14 Family used QPL
Yeh et al^41^ Pall. Care	USA	30		77% Requested complete information (good and bad); 83% requested medical information	↑ Satisfaction ↓ Anxiety (*P* < .005) 90% helpful and relevant; 97% easy to understand
McLawhorn et al^44^ Prognostic focused	USA	128 (CG) 166 (IG)			↑ Do not resuscitate IG (vs CG) ↑ Hospice referral rate IG (vs CG)
Walczak et al^12^ End of Life care	Australia and USA	15 (Au) 11(USA)			55% Intent to use QPL 45.16% did not wish to discuss life‐expectancy Q.
Berger et al^42^ Outpatient Care	USA	90 (P) 15(Cl)		36% Used the tool 31% discussed with Cl	49% helped prepare for follow‐up 74% (Cl) identified areas of concern
Jayasekera et al^46^ 21 gene RS testing	USA	136 (Study 1) 65 (Study 2)	98.3% Easy to understand 88.5% enough time to read	82% QPL useful 61.5% asked additional question	↓ Decision conflict (*P* < .01) ↑ Overall knowledge (*P* < .01) ↑ Treatment knowledge (*P* < .01)

#### 2.3.3 Effectiveness of the QPL

Using randomized control trials, psycho‐oncology research has tested the effectiveness of the QPL based on patients' self‐reported outcomes at three different time points: (i) before the consultation; (ii) soon after the consultation; and (iii) on follow‐up. The effectiveness of the QPL was measured using the following patient outcomes: (i) communication (ie, patients' question‐asking, amount of information given, and length of the consultation); (ii) psychological (ie, levels of anxiety, depression, and patient satisfaction); and (iii) cognitive (ie, patients' recall of information exchanged during the consultation).^26^ Table 3 provides a detailed description of intervention studies using the QPL.

**TABLE 3:** Overview of intervention studies on QPL to understand the effectiveness of the QPL

Study	Groups compared	Country	n	Effectiveness of the QPL							
				Communication				Psychological			Cognitive
				Patient preference	Q. asked	Utility	Counsult. time	Anxiety	Psycho‐ adjustment	P. satisf	Recall
Butow et al^1^	QPL; general info sheet	Australia	142		QPL (vs CG), *ns* ↑ Prognosis Q. (35%)		*ns*	*ns*	*ns*	*ns*	*ns*
Brown et al^33^ Med	QPL and coaching; QPL alone; stand. care	Australia	QPL (20) QPL+ coach (20) CG (20)		↑IG (vs CG), *ns*↑Tests category, *P* = .048			*ns*	*ns*	*ns*	
Brown et al^2^ Med & Rad	QPL+ Pro D; QPL+ Pass D; Standard care (CG)	Australia	QPL+ Pro D (81) QPL+ Pass D QPL (79) CG (158)	*ns*	IG (vs CG), *ns* ↑ Prognosis Q., *P* = .03		↓QPL + Doc (vs QPL & CG) $\bar{X}$= 28.50 min, SD = 9.87	↑ Post‐ consult in QPL (vs QPL + doc & CG), *P* = .004 Follow‐ up; *ns*		*ns*	↑ QPL + doc (vs QPL), *P* = .036 CG vs IGs; *ns*
Bruera et al^39^ Breast C	QPL; general infor	USA	IG (30) CG (30)		↑ Diagnosis Q. IG (vs CG) (2.5 vs 1.4), *P* = .025	↑ IG(vs CG) (7.9vs 5.7), *P* = .01	*ns*			*ns*	
Butow et al^9^	CCPP (include QPL); CG	Australia	IG (80) CG (84)	*ns*; 87% participants preferred info	↑ CCPP (vs CG); (13 vs 9), *P* = .009 ↑ Prognosis, *P* = .001	↑ family involved (*P* = .004)		↑ Before consult; CCPP vs CG (*P* = .04) Follow‐up; *ns*		*ns*	
Clayton et al^3^ Pall. Care	Physician endorsed QPL; standard care	Australia	IG (92) CG (82)		↑ QPL (vs CG), *P* < .001 ↑ Pall. Care, *P* < .001 ↑ Lifestyle, p = .03 ↑ Support, *P* = .05 ↑ Prognosis, *P* = .05	95% patients 46% physician	↑ in QPL(vs CG) (37.5 min vs 30.5 min), *P* = .002	Post consult (24 hours); *ns* Follow‐up; *ns*		↑ Overall	
van Weert et al^13^	Intervention (QPL); control group	Netherlands	Pretest: IG (64) CG (51) Posttest: IG (55) CG (40)		↑ IG (vs CG), *P* < .05 ↑ Treatment Q., hospital routine Q. and side‐effects Q.		*ns*				↑ IG (vs CG), *P* < .10
Lim et al^10^ Br.C & Abdom	QPL; CG	Singapore	IG (114) CG (116)			↓useful (B = ‐0.8) ↓aid comm (B = −1.6)		↓QPL (vs CG) in Time 4 (follow‐up), *P* = .010			
Shirai et al^40^ Advanced cancer	QPS + information sheet; standard care	Japan	IG (32) CG (31)		*ns*	↑ IG (vs CG); *P* = .033				↑ both group; Diff; ns	
Tattersall et al^47^ Clinical Trial	QPL; CG	Australia	IG (45) CG(43)			QuIC: *ns*		*ns*		*ns*	
Eggly et al^48^ Racial disparity	QPL only; QPL + Comm coach; Standard care	USA	QPL (42) QPL+ coach (36) CG (44)		↑ QPL only arm (vs standard care) *P* = .02 QPL + coach (vs CG), *ns*	↑ M (2.80) in both IG and CG; *ns*	*ns*				
Rodenbach et al^30^	QPL coaching; standard care	USA	IG (84) CG (86)		↑ QPL related topics IG vs CG (= 1.7) *P* < .001 ↑ Prognosis Q. IG (vs CG), *P* = .2		*ns*				
Bouleuc et al^49^ EOL	QPL; CG	France	IG (71) CG(71)		↑IG (vs CG), *P* = .03 ↑Pall. Care IG (vs CG), *P* = .012 ↑EOL IG (vs CG), *P* = .018	80% QPL useful 90% easy to understand		No effect IG (vs CG), *P* = .05		↑ Satis Phy Tech S, *P* = .024	
Zetzl et al^50^ Radiation	QPL; CG	Germany	IG (139) CG(140)			60.4% frequent use 55% very helpful				↑ iE‐Q, IG(vs CG) *P* = .007	

##### Communication outcomes

Research using the QPL with cancer patients has indicated the number of questions asked during the medical consultation was higher in the intervention as compared to the control group.^3^, ^9^, ^33^, ^48^, ^49^ Interestingly, patients using the QPL asked questions on specific topics such as diagnosis, prognosis, lifestyle changes, and quality of life (QoL),^1^, ^3^, ^33^, ^39^ suggesting QPL use may have helped patients to confidently think about and engage in communication on topics important to them. The findings regarding length of consultations where QPLs are used have been mixed. For example, Clayton et al^3^ reported that patients who used the QPL had a longer consultation than the control group because the former may discuss more issues during the consultation than the latter. However, Brown et al^2^ found consultations were shorter when the oncologist promoted the use of the QPL during the consultation. The authors suggested this may be because using the QPL helped patients to prepare for their consultation by clarifying questions, and physicians formally addressing the questions may increase communication efficiency, avoiding circuitous discussions with the patient.^2^ Contradicting these findings, other studies showed no differences in the consultation time between QPL group and standard care group,^13^, ^39^, ^48^ suggesting a QPL does not put a strain on the time for or cost of care.

##### Psychological outcomes

Psychological outcomes frequently measured in research using QPL interventions are anxiety, depression, and patient satisfaction. Clayton et al^3^ reported anxiety was similar in both the QPL and control groups at 24 hours and at follow‐up 3 weeks later for advanced cancer patients in palliative care. Interestingly, Brown et al^2^ reported oncology patients who used the QPL and were paired with a passive physician (ie, not promoting the QPL) reported higher levels of anxiety as compared to patients who did not receive the QPL (ie, control group) and patients who used the QPL while paired with a proactive physician (ie, promoted the QPL).^2^ The authors suggested this may be because physician endorsement of the QPL helped patients raise difficult questions, thus reducing levels of anxiety.

In terms of the psychological outcomes of both depression and patient satisfaction, there were no differences between patients who used the QPL (ie, intervention) and those who did not (ie, control). These findings remained unchanged during the postconsultation and follow‐ups.^2^, ^3^, ^9^, ^33^, ^49^ Butow et al^9^ suggest this may be due to both the patient groups' (control and intervention) low levels of depression at baseline, meaning, there was little room for change in depression scores. Similarly, QPL studies examining patient satisfaction reported it remained unchanged for both the intervention and control groups.^1^, ^2^, ^3^, ^47^ Indeed, most patients reported higher levels of satisfaction irrespective of the study arm.^3^, ^11^, ^40^ Brown et al^33^ suggested this may be explained with cognitive dissonance theory,^51^ wherein individuals experiencing discordance in their cognitions (ie, beliefs, values, opinions, attitudes) and behaviors will seek to restore consistency by reducing the importance of the discordant beliefs, adding more accordant beliefs which will outweigh the discordant beliefs, or changing discordant beliefs to avoid inconsistency. In this way, the authors posited patients may associate feelings of dissatisfaction with a lack of trust in the physician, which can be problematic dealing with a life‐threatening illness such as cancer.^33^ Additionally, the authors argued patients with cancer relied on oncologists' knowledge and expertise far more than patients with less critical illnesses, making cancer patients more likely or inclined to overlook characteristics they considered unsatisfactory in their oncologists.^33^

Interestingly, recent research exhibits contrasting findings to the above studies: Bouleuc et al^49^ found that patients in the QPL group expressed greater satisfaction with the physician's technical skills than their counterparts, and Zetzl et al^50^ reported that patients in the QPL group had higher scores on perceived interaction with the medical team than their counterparts. These findings suggest a change in trends of patients' expectations and needs from their physicians, thus necessitating a continued examination of the psychological outcomes of using the QPL.

##### Cognitive outcomes

QPL research in psycho‐oncology has also assessed the cognitive outcome of recall of information discussed during the patient‐physician interaction after the consultation. The ability to recall information was evaluated based on how much treatment‐related information patients were able to recall soon after their consultation. Butow et al^1^ assessed recall of information during a short‐term follow‐up (4‐20 days after the consultation) and found no improvement with QPL use. Interestingly, Brown et al^2^ observed an increase in recall of information when the physicians were actively involved in the consultation and systematically reviewed the questions in the QPL. This finding suggests that phyisicans who supported question‐asking and responded to the issues raised through the QPL and reinforced the treatment information shared with their patient which increased patients' ability to recall this information.

## 3 A CASE FOR THE QPL IN THE INDIAN ONCOLOGY SETTING

### 3.1 The oncology care scenario in India

In India, 11 57 294 new cancer cases and 7 84 821 deaths due to cancer were reported for the year of 2018.^52^ While the Government of India's efforts to increase cancer screening is reducing this mortality rate, it has led to increased incidence, further challenging the already insufficient healthcare resources in the nation.^53^ A major resource deficit in India is the physician to patient ratio (0.77:1000) as compared to the World Health Organization recommendation of 1:1000.^14^ This gap hinders access to quality healthcare in India,^15^ resulting in decreased time with the physician during medical consultations (mean time: 1.5‐2.3 minutes).^54^ This scarcity of time has several consequences such as decreased patient understanding of their illness,^55^ reduced satisfaction,^56^ and a poor physician‐patient relationship.^57^ Interestingly, recent research in India shows physicians become dissatisfied when they are unable to provide their patients with adequate time and attention.^58^ An obvious solution to these issues may be the introduction of communication skill training (CST) for physicians which is focused on effective information‐giving and empathy.^59^, ^60^ Even so, introducing CST in Indian oncology settings may not improve patient‐physician communication for several reasons. First, with the already mentioned low physician‐to‐patient ratio, it may be futile since physicians will continue to be hard‐pressed for time and may not be able to cater to patients' information and emotional needs beyond what they currently do. Second, it may be difficult for the physician and patient alike to embrace an altered form of physician‐led communication when patients expect and desire their physicians to play an authoritarian role.^61^

Therefore, a practical solution for the Indian oncology setting is patient‐/caregiver‐led communication interventions such as the QPL which help structure medical consultations, allow patients/caregivers to think about the questions/concerns most important to them, understand the type and range of questions about the illness, reduce consultation times, and may become a valuable tool in the medical decision‐making process.^29^ Indeed, a QPL may be effective to empower Indian patients/caregivers with essential communication skills.

### 3.2 Family and oncology care in India

In India, family members are an integral part of the illness experience and play a central role in diagnosis and management of a chronic illness such as cancer.^62^, ^63^ However, family support can be both helpful and unhelpful to the patient. It can benefit the patient by reducing the burden of medical decision‐making and providing financial and emotional support.^4^, ^62^, ^63^ On the other hand, family involvement during the illness can result in collusion, selective sharing of information with the patient, and nondisclosure of the diagnosis which may hinder patients' well‐being.^1^, ^64^, ^65^ Through their study in South India, Harding, Nair, and Ekstrand^66^ reported the long‐lasting impact of cancer nondisclosure to the family in terms of lost employment and increased debts due to medical costs. The authors highlight families' lost opportunities to talk about their patient's psychological and spiritual needs due to collusion,^*^ suggesting suboptimal use of healthcare services and family support.

These issues notwithstanding, families continue to play a crucial part throughout the illness trajectory in India.^4^, ^63^ Therefore, harnessing the family positively to contribute to patient well‐being is imperative. One important way families can facilitate and improve patient well‐being is through involving and supporting the latter in the communication exchange with their treating physician. Highlighting this, in a study where the QPL was used by patients undergoing palliative care and their caregivers, Clayton et al^3^ found that not only did the QPL help the latter ask questions regarding their caregiving issues but also helped patients and caregivers raise difficult topics such as prognosis and facilitated a discussion between the patient and physician. In India, it is possible QPLs will not only help caregivers navigate sensitive topics with patients and physicians but provide them insights into the kind of questions patients may have about their illness. Indeed, a QPL for family caregivers may unlock opportunities for meaningful, truthful, and open communication between patients and their families.

### 3.3 Patients' unmet information needs in India

Recent trends in India highlighted that patients actively seek information and express the need to be involved in their medical decision‐making.^4^ Despite this desire for active participation, patients in India have consistently reported dissatisfaction especially with regard to the information provided to them.^4^ The key explanations for patients' unmet information needs are family filtering “harmful” or demoralizing information,^63^ an unequal patient‐physician relationship,^64^ insufficient time with the physician,^68^ and the need to hear bad news in the company of another trusted adult.^4^ This hindrance to information resulted in Indian patients reporting increased levels of anxiety, depression, worries, and dissatisfaction with their care.^69^, ^70^ Since the increase in internet access and use in India,^71^ patients have addressed their information needs through using the internet. However, internet use has inherent issues such as leading patients to access information which is incorrect or inappropriate and giving rise to a problematic patient‐physician relationship.^72^ Consequently, it is important to address patients' unmet information needs during the medical consultation, thus ensuring they receive accurate information about their illness and treatment from their treating physician.

In this scenario, a QPL presents multiple beneficial opportunities to Indian cancer patients. First, it can empower patients to formulate, organize, and ask questions about their illness and treatment. Second, as discussed in the previous sections, QPLs are more likely to effectively address patients' concerns and questions as they are developed for this purpose. Third, since QPLs are generated by patients for patients, they can be reliable and genuine tools for improving patients' knowledge about their illness. Finally, by addressing patients' issues arising from the lack of access to information, QPLs can help reduce levels of psychological distress and increase patient satisfaction.

## 4 STRENGTHS AND LIMITATIONS

The strengths of the review are that it provides a comprehensive overview of QPL in the oncology setting, highlighting the poor patient‐physician ratio and integral role of the family caregivers in cancer communication in India, and it suggests how QPL can address the gap in communication in a culturally sensitive manner.

The review has some limitations. First, it limits literature to an oncology setting. Past research has shown the QPL can be used in other illness contexts (eg, gynecological issues,^22^, ^23^ chronic kidney disease,^24^ attention‐deficit/hyperactivity disorder^25^). Therefore, future reviews can explore the applicability of the QPL across different illnesses in India. Second, a QPL can be resource intense to develop (owing to the several steps involved before implementation) and is typically focused on a specific aspect of the illness and treatment journey (eg, type of cancer, treatment, and palliative care). However, the current review did not consider this issue which can be of key importance in resource compromized settings as found in India. QPL research in India should take into account this aspect and identify appropriate resources to fulfil these steps or examine methods to expedite the development of a QPL.

## 5 CONCLUSION

Research in India indicates changing trends in patient‐physician communication with patients expressing a need to be actively involved in their treatment and medical decisions. A key method to engage in one's illness decisions is to become acquainted with relevant medical information. However, a poor physician‐patient ratio and family involvement throughout the medical care trajectory are primary contributors to Indian patients' unmet information needs. A QPL can help address these issues by providing both patients and their caregivers an opportunity to ask questions about the illness and its treatment and assisting patients' active involvement. Additionally, the QPL helps patients to be systematic in seeking difficult information, thus addressing issues centered on the strained medical resources in India. Therefore, a QPL may be an appropriate tool for facilitating communication between the oncologist, patient, and family caregiver in India.

## ETHICAL STATEMENT

Not applicable.

## CONFLICT OF INTEREST

There is no conflict of interest.

## AUTHOR CONTRIBUTIONS

All authors had full access to the data and take responsibilities and the accuracy of data analysis. *Conceptualization*, S.C., M.C.; *Methodology*, S.C., M.C., H.D., P.B.; *Investigation*, S.C., S.M.; *Writing ‐ Original draft*, S.C., S.M.; *Writing ‐ review and editing*, M.C., S.C., S.M., H.D., P.B; *Supervision*, M.C., H.B., P.B.

## Data Availability

Data sharing not applicable to this article as no datasets were generated or analyzed during the current study.

## Endnotes

*
Collusion refers to a secret agreement between the medical staff and the caregivers to withheld or not share information (diagnosis, prognosis or patients medical details) among the patients. The medical information maybe selectively or completely not disclosed to the patient or their relatives.^67^

†
Butow et al^1^; Brown et al.^33^

‡
Individuals with clinical experience.

^§^

Expert Panel consisted of three former employees, two clinical geneticists, a genetic nurse, psychologist and a social scientist.

¶
Brown et al^33^; Bruera et al^39^; Butow et al.^1^

#
Walczak et al.^32^
